# Gene Delivery by Subconjunctival Injection of Adenovirus in Rats: A Study of Local Distribution, Transgene Duration and Safety

**DOI:** 10.1371/journal.pone.0143956

**Published:** 2015-12-07

**Authors:** Guei-Sheung Liu, Jiang-Hui Wang, Jia Hui Lee, Pei-Jhen Tsai, Han-En Tsai, Shwu-Jiuan Sheu, Hsiu-Chen Lin, Gregory J. Dusting, Ming-Hong Tai, Youn-Shen Bee

**Affiliations:** 1 Centre for Eye Research Australia, Royal Victorian Eye and Ear Hospital, East Melbourne, Victoria, Australia; 2 Ophthalmology, Department of Surgery, University of Melbourne, East Melbourne, Victoria, Australia; 3 Department of Ophthalmology, Kaohsiung Veterans General Hospital, Kaohsiung, Taiwan; 4 Institute of Biomedical Sciences, National Sun Yat-Sen University, Kaohsiung, Taiwan; 5 School of Medicine, National Yang-Ming University, Taipei, Taiwan; 6 National Defense Medical Center, Taipei, Taiwan; 7 Yuh-Ing Junior College of Health Care & Management, Kaohsiung, Taiwan; University of Florida, UNITED STATES

## Abstract

Subconjunctival injection is a minimally invasive route for gene delivery to ocular tissues, but has traditionally been limited to use in the cornea. The accurate ocular distribution of virus has not, however, been previously investigated. Adenovirus is an attractive gene vector as it can deliver large genes and allow for short-term gene expression, but how safe it is when delivered via subconjunctival injection remains to be established. We have characterized the bio-distribution and safety of subconjunctivally administered adenovirus in Brown Norway rats. The bio-distribution and transgene duration of adenovirus carrying luciferase gene (Ad-Luci) at various time intervals were evaluated via bioluminescence imaging after subconjunctival injection. Adenovirus carrying a reporter gene, β-galactosidase (Ad-LacZ) or hrGFP (Ad-hrGFP) was administered subconjunctivally and the viral distribution in various ocular tissues was assessed by histological analysis and quantitative PCR (qPCR). Hepatic damage was assessed by biochemical and immunohistological analysis with TUNEL stain. Systemic immunogenicity was assessed by measuring serum level of TNF-α via ELISA, 2 hours and 14 days after administration of adenovirus. Retinal function was examined by electroretinography. Subconjunctival injection of Ad-Luci induced luciferase expression in the injected eyes within 24 hours, for at least 64 days. Histological analysis showed adenovirus distributed across anterior and posterior ocular tissues. qPCR demonstrated different amounts of adenovirus in different ocular tissues, with the highest amounts closest to the injection site Unlike the intravenous route, subconjunctivally delivered adenovirus did not elicit any detectable hepatic injury or systemic immunogenicity. Retinal function was unaffected by adenovirus irrespective of administration route. In conclusion, an adenoviral vector administered subconjunctivally can infiltrate into different ocular tissues and lead to short-term ocular transgene expression, without causing hepatic injury and immune activation. Therefore, subconjunctivally administered adenovirus may be a promising gene delivery approach for managing anterior and posterior segment eye diseases requiring short-term therapy.

## Introduction

Gene therapy is an emerging and powerful modality in the treatment of eye diseases. It is unique in its ability to manipulate the expression or coding of a dysfunctional gene, allowing correction of the underlying pathological mechanism of the disease with prolonged benefits. [1] In recent experimental studies, gene therapy has been shown to have exciting therapeutic potential in many ocular applications, including treatment of currently incurable genetic eye diseases, as well as providing more effective treatments for common diseases affecting anterior and posterior segments of the eye.

Successful gene therapy is particularly dependent upon the suitable vector and route of administration that ideally has low toxicity, a high safety profile, and results in efficient therapeutic gene expression of the therapeutic gene product in target cells [1]. Currently, Adeno-Associated Virus (AAV) is the most commonly used vector in registered gene therapies for ocular disorders, but it lacks the ability to carry larger genes and are not ideal for situations when only short-term gene expression is desired.

Adenovirus is an attractive viral vector as it can produce large amounts of highly purified recombinant virus, which efficiently infects a wide variety of dividing and non-dividing cells [2, 3]. It can also deliver a large-sized foreign gene of up to 10kb. These features make adenovirus a suitable vector for delivering genes to target sites both *in vitro* and *in vivo*, and indeed, its development has been explored in different models of ocular disease, such as choroidal or retinal neovascularization, retinoblastoma, glaucoma and corneal injury [1, 4–8]. Moreover, the expression profile of adenovirus has been shown to have potential in situations where short-term gene therapy is desired. To date, there have been limited studies on the safety profile of adenovirus and its systemic effects.

Additionally, most registered gene therapies use intravitreal injection to deliver viral vectors [9]. This route of administration is invasive and has significant risks. On the other hand, intravenous administration of gene therapy may be less invasive, but results in more adverse systemic effects. Subconjunctival injection is a route of administration that has been shown to effectively deliver gene therapy to the eye, and has been shown to have less adverse effects when compared to systemic or topical gene delivery [10–13]. However, few studies have accurately characterized the local bio-distribution and amount of subconjunctival gene delivered in the different portions of the eye, and as such, the use of subconjunctival injection as a mode of gene therapy delivery has been largely limited to ocular diseases affecting the cornea.

Therefore, in this study we characterized the local bio-distribution of genes delivered by subconjunctival injection of adenovirus in different parts of the eye in rats. In particular, we were interested in investigating whether route of gene delivery could be effective in the posterior segment of the eye, such as retina and choroid, which would have significant implications for its potential use in ocular diseases affecting these areas. We also characterized the safety profile of this mode of gene delivery, including local and systemic immunogenicity and toxicity, when compared to intravenous administration, in order to determine its feasibility for use in the clinical setting.

## Materials and Methods

### Construction and production of recombinant adenovirus

The recombinant adenovirus encoding luciferase (Ad-Luci), β-galactosidase (Ad-LacZ), and humanized recombinant green fluorescent protein (Ad-hrGFP) were applied in this study. The plasmids of pShuttle-CMV-LacZ and pShuttle-IRES-hrGFP-2 were from a commercial kit for adenoviral production (AdEasy Adenoviral Vector Systems, Stratagene, CA, USA). Luciferase DNA was digested with KpnI prior to cloning into a vector, pShuttle-CMV (Stratagene), which ultimately yielded the plasmid of pShuttle-CMV-Luciferase. All recombinant adenovirus were generated in 293A cells (Invitrogen, CA, USA) using AdEasy Adenoviral Vector System (Stratagene). After homologous recombination, the virus plaques were verified by checking for cytopathic effect and PCR as previously described [14]. The virus was amplified and purified with cesium chloride under ultracentrifugation and then desalted by G-25 gel-filtration chromatography. The number of viral particles and titer were determined by measuring the optical density at 260 nm and plaque-forming assay on 293A cells before storage at -80°C.

### Animal care and adenovirus delivery by subconjunctival or intravenous administration

All experimental procedures were designed and performed on rats and were approved by the institutional animal care and use committees (St. Vincent's Animal Ethics Committee and Kaohsiung Veterans General Hospital’s Animal Ethics Committee) to ensure that the animals did not suffer unduly during and after the experimental procedure. No rats became ill prior to the experimental endpoint. The health and well-being of the rats were monitored. Minor stress induced by operation was looked for daily by observing the motility, grooming and feeding behavior after eye injections. Typical behaviors that indicate discomfort include agitation and excessive rubbing of the eye.

Male Brown Norway pigmented rats weighing between 150g and 200g were used in this study. The animals were supplied by Animal Resources Centre (Perth, Australia) and National Animal Center (Taipei, Taiwan), and animals were housed at the EMSU rat facility and Kaohsiung Veterans General Hospital’s Animal Center. All rats were housed in standard cages, two rats per cage, free access to food and water under a 12 hours light (50 lux illumination) and 12 hours dark (<10 lux illumination) cycle with a temperature controlled environment to minimize possible light induced damage to the eye.

Prior to adenovirus delivery by subconjunctival and intravenous injection, animals were anaesthetized by intraperitoneal injection of a mixture of ketamine (70mg/kg) and xylazine (6.5mg/kg) in phosphate-buffered saline (PBS). Topical local anesthesia, proxymetacaine 0.5% drops, was given to rats prior to subconjunctival injection to provide local anaesthesia. Under dissection microscope, a small incision was made through the conjunctiva at the superotemperal quadrant with a 33-gauge needle attached with 1ml syringe, and 50 μL of adenovirus or the PBS was injected in both eyes for duration assay or in one eye for bio-distribution and safety assy. For intravenous injection, each rat was administered with 50 μL of adenovirus through tail vein. After injection, the rats were monitored included two hourly checks for the first 6 hours then twice daily for the following 3 days. All animals were then sacrificed at the desired day using Lethobarb (200 mg/kg, intraperitoneal injection) as per each experiment after the single subconjunctival injection, and tissues were harvested and for further assessment (S1 Fig).

### Bioluminescence imaging

To perform the bioluminescence imaging, rats were anaesthetized by intraperitoneal injection of the combination of ketamine (70mg/kg) and xylazine (6.5mg/kg) supplemented with a topical anaesthetic agent (proxymetacaine 0.5%) and received a single injection of D-luciferin (20 mg/mL; Promega, WI, USA) through intravenous (100 μL for intravenous injection group) or subconjunctival (50 μL for subconjunctival injection group) route. *In vivo* luciferase activity of the rats received Ad-Luci via subconjunctival or intravenous injection was recorded by IVIS Imaging System (200 Series, Caliper Life Sciences, MA, USA) at day 1, 2, 7, 14, 28, 35 and 64 after injection. A gray scale image of body surface was obtained in the chamber under dim illumination followed by a 5 minutes acquisition and an overlay of the pseudocolor images. The images of spatial distribution were captured and bioluminescence intensity of tissues was quantitated by the software.

### Histological analysis

Rats were anesthetized and subjected to a single subconjunctival injection with 50 μL of Ad-LacZ (1x10^10^ GC/eye). Eyes were enucleated after 7 days of injection and were immediately processed to sections embedded with paraffin for further staining. The X-Gal staining was then applied on the sections to find out the distribution of Ad-LacZ in eyes. The paraffin sections were fixed with 4% paraformaldehyde for 15 minutes at room temperature and were washed twice with cold PBS. The slides were incubated with stain solution containing 1.3 mM MgCl_2_, 15 mM NaCl, 44 mM HEPES buffer, 3 mM K_3_FeCN, 3 mM K_4_FeCN, 0.5 mg/mL X-gal in distilled water. The slides were rinsed twice with PBS and were counterstained with eosin to examine the LacZ positive cells and tissue morphology.

### Quantitative PCR for viral genomic DNA

Seven days after administered with Ad-hrGFP or PBS via subconjunctival injection, the rat eyes were dissected to the corneal, retina, choroid and sclera, respectively. Total genomic DNA from each type of tissue was extracted and purified using commercial kit in accordance with the manufacturer's instructions (DNease Blood & Tissue Kit; Qiagen, Victoria, Australia). Briefly, tissue was homogenized in the lysis buffer and total DNA was extracted and purified with a column system. To calculate the copy numbers of the Ad-hrGFP in different tissues, a standard curve of 10-fold serial dilution using linear pShuttle-IRES-hrGFP-2 was established (S2 Fig). The total DNA (100ng) was then amplified by quantitative PCR (7300 real-time PCR systems; Life Technologies) using fast SYBR green master mix (Invitrogen, CA, USA), along with the hrGFP forward primer (5´ CTCACGGGGATTTCCAAGTC 3´) and the hrGFP reverse primer (5´ ATGCAGTCGTCGAGGAATTG 3´). The Ad-hrGFP genome copies in tissues were then calculated based on the standard curve. To generate the standard curve, the plasmid of pShuttle-IRES-hrGFP-2 was digested by restriction enzyme XhoI (NEB, MA, USA) to form the linear DNA. The linear DNA was then purified with QIAquick PCR Purification Kit (Qiagen) and was 10-fold serially diluted, ranging from 1x10^9^ to100 DNA copies per 5μL of dilution. Standard curve was used to calculate the genomic DNA copies of test samples, which was denoted as genome copies (GC) per μg of total DNA of differential ocular tissues. The viral genome copies were assumed that one viral vector contained one viral genome copy.

### Gene expression detected by quantitative PCR

Total RNA from different tissues were exacted and purified using commercial kits in accordance with the manufacturer's instructions (RNeasy Mini Kit; Qiagen). Briefly, each tissue in the lysate was homogenized, and total RNA was purified with a column system. The total RNA (100ng) was then reverse-transcribed to cDNA using a high-capacity RT kit (catalogue No. 4374996; Life Technologies, Victoria, Australia). Quantitative PCR was performed using either a SYBR Green master mix with the hrGFP primer set or using a TaqMan Universal PCR master mix and commercially available probe and primer sets (TaqMan Gene Expression Assay, Life Technologies) for rat TNFα (Rn99999017_m1). Rat Actb (Rn00667869_m1) was used as a reference gene.

For analysis of mRNA expression, relative expression levels of TNF-α in different tissues between rats administered Ad-hrGFP and PBS were calculated using ΔΔCt method which has been described by Livak previously [15]. In comparison with the rats administrated PBS, tissues of the rats administered Ad-hrGFP containing more than two fold of TNF-α mRNA were defined as positive.

### Biochemical parameter (liver function) analysis

Arterial blood was drawn from rats administered Ad-Luci via either subconjunctival or intravenous injection before injection and 14 days after injection. Samples were centrifuged at 15000 rpm for 15 min for serum collection. Serum level of glutamic oxaloacetic transaminase (GOT) and glutamic pyruvic transaminase (GPT) were measured by an autoanalyzer (Vitros 350 chemistry analyzer, Johnson & Johnson, MN, USA).

### Terminal deoxynucleotidyl transferase-mediated dUTP-biotin nick-end labeling (TUNEL) assay

The liver tissue from rats which had received Ad-Luci injection was fixed in 4% paraformaldehyde overnight and embedded in paraffin using standard histological procedure [16]. Terminal deoxynucleotidyl transferase-mediated dUTP-biotin nick-end labeling (TUNEL) stain was performed in accordance with manufacturer's protocols (Roche, Penzberg, Germany). Briefly, Paraffin-embedded sections were deparaffinized, rehydrated, and were subjected to antigen retrieval with 0.1M citrate buffer (Sigma-Aldrich, MO, USA). The sections were incubated with working-strength TdT buffer for 1 hour at 37°C. After washing with PBS for three times, the sections were incubated with 4', 6-diamidino-2-phenylindole (DAPI; Sigma-Aldrich) at room temperature for 10 minutes. After washing with PBS once, the sections were fixed in mounting medium (Dako, Denmark). TUNEL-positive cells were detected by an Olympus BX61 microscope under 200x magnification.

### Cytokine analysis by ELISA

The serum level of interleukin-1β (IL-1β) and cytokine-induced neutrophil chemoattractant factor-1 (CINC-1) from rats administered Ad-Luci were measured by enzyme-linked immunosorbent assay (ELISA; R&D system, MN, USA) kit according to manufacturer’s instruction.

### Electroretinogram (ERG)

The single bright flash white electroretinograms (ERG; UTAS-E 300; LKC Technology, MD, USA) under the dark-adapted environment was performed to assess the effects of adenoviral vector administered by intravenous or subconjunctival injection on retinal function before injection and day 14 after the injection. After at least 30 minutes of darkness adaptation, rats were anaesthetized and gold foil was placed on the cornea with 2% methylcellulose gel application (Omni Vision, Neuhausen, Switzerland). A reference electrode was attached to the shaved scalp and a ground electrode was clipped to the rat’s ear. After reducing the background noise below 60 Hz, a single flash of bright light (duration, 100 ms), 30 cm away from the eye, was used as the light stimulus. The responses were amplified with a gain setting 500 μV and were filtered with an amplifier (low, 0.3 Hz; high, 500 Hz). Data were acquired, digitized, and analyzed using EM for Windows, version 2.6.

### Statistical Analysis

Data are expressed as mean ± standard error of the mean (SEM). Mean data were analyzed with unpaired t-tests or two-way analysis of variance (ANOVA) followed by post-hoc Tukey analysis (SPSS software, version 18.0 or GraphPad Prism 6.0). A value of p<0.05 was regarded as statistically significant.

## Results

### Adenoviral transgene expression in the eye via subconjunctival injection

**Fig 1:**
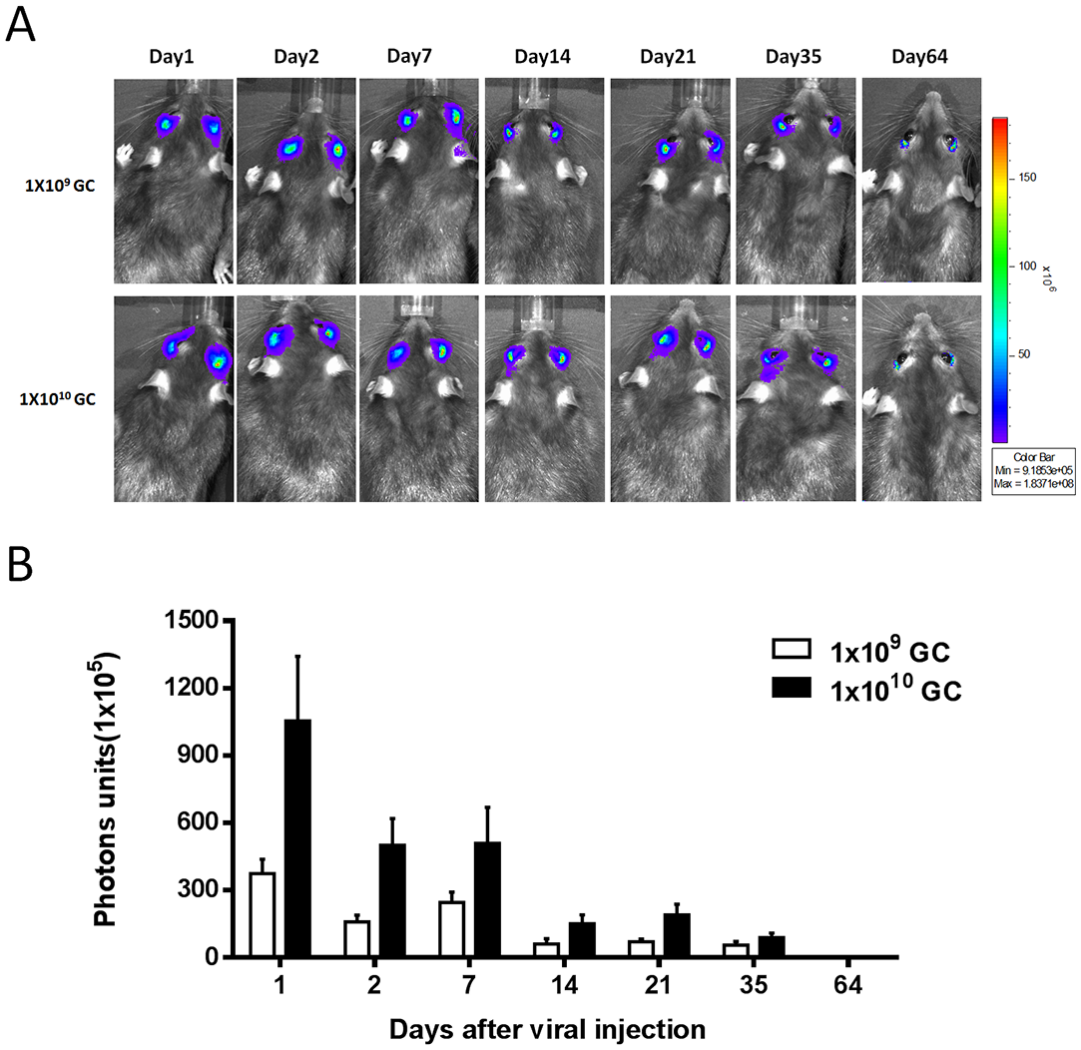
Viral dosage and duration of adenovirus-mediated gene delivery after a subconjunctival injection in the rats. (A) Bioluminescence images of rat eyes at day 1, 2, 7, 14, 28, 35 and 64 after a subconjunctival injection of Ad-Luci (1x10^9^ and 1x10^10^ GC/eye). (B) Luciferase activity was measured in unit of photons by bioluminescence analysis. Data are presented as the means ± SEM (n = 5).

**Fig 2:**
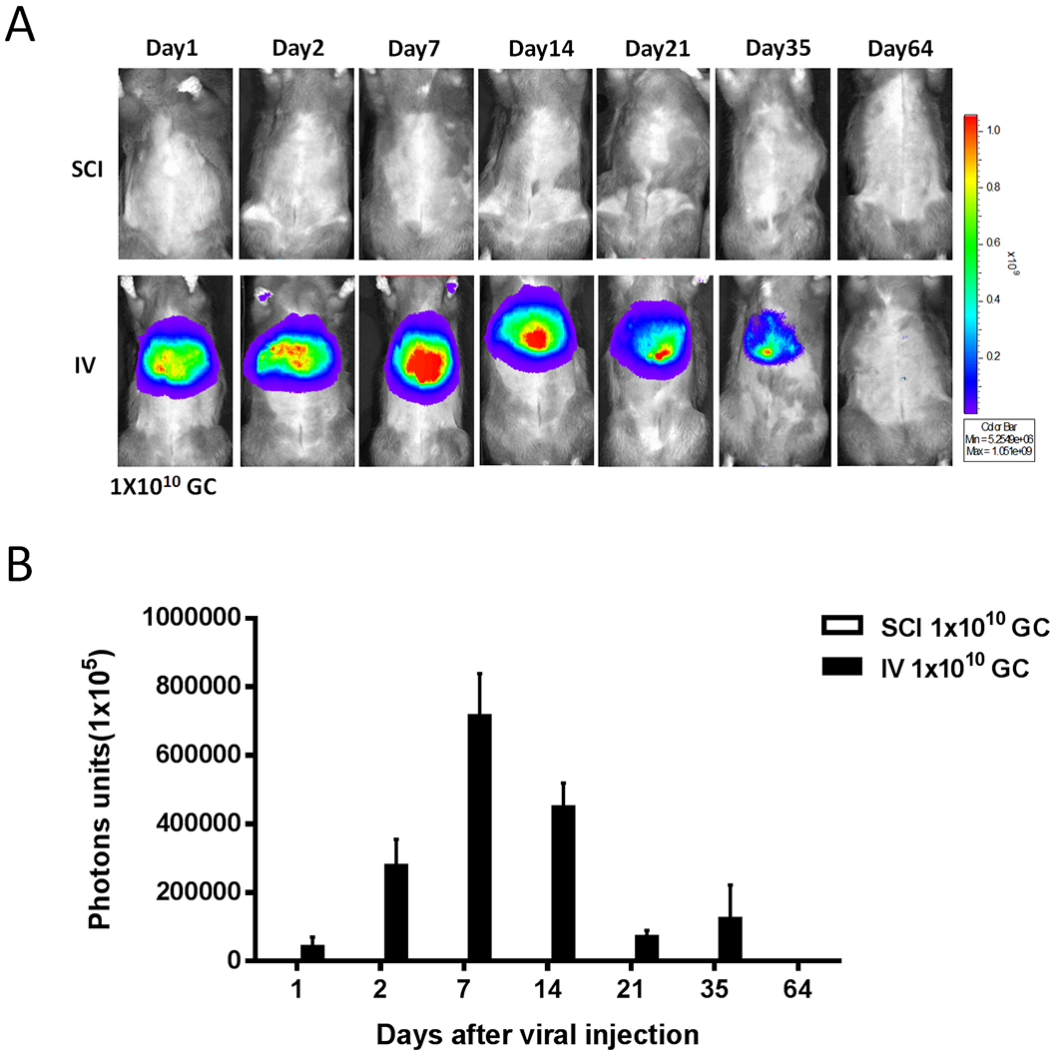
Bio-distribution of adenovirus after a subconjunctival or intravenous injection in the rats. (A) Bioluminescence images of rat at day 1, 2, 7, 14, 28, 35 and 64 after a subconjunctival or intravenous injection of Ad-Luci (1x10^10^ GC/eye). (B) Luciferase activity was measured in unit of photons by bioluminescence analysis. Data are presented as the means ± SEM (n = 5).

To evaluate the viral dose and duration of transgene delivered after subconjunctival administration of adenoviral vector, we investigated luciferase transgene expression in mice after injection of adenovirus encoding luciferase (Ad-Luci) by bioluminescence analysis. Twenty-four hours after injection, the expression of luciferase significantly increased in both groups of 1x10^10^ GC/eye and 1x10^9^ GC/eye injections, and localized mainly around the injection site of the eye, but sustained for up to 64 days (Fig 1). This result is consistent with the known expression profile of a transgene given via adenoviral gene delivery [17]. Importantly, apart from the eye, there was no bioluminescence detected in other organs of the rat receiving Ad-Luci through conjunctiva, particularly in the liver. In contrast, there was a significant increase of bioluminescence in the liver of rats administered Ad-Luci via intravenous injection (1x10^10^ GC/eye; Fig 2). The results suggested that the adenovirus via subconjunctival injection leads to local and rapid expression of the transgene in the eyeball, with no expression in other organs.

**Fig 3:**
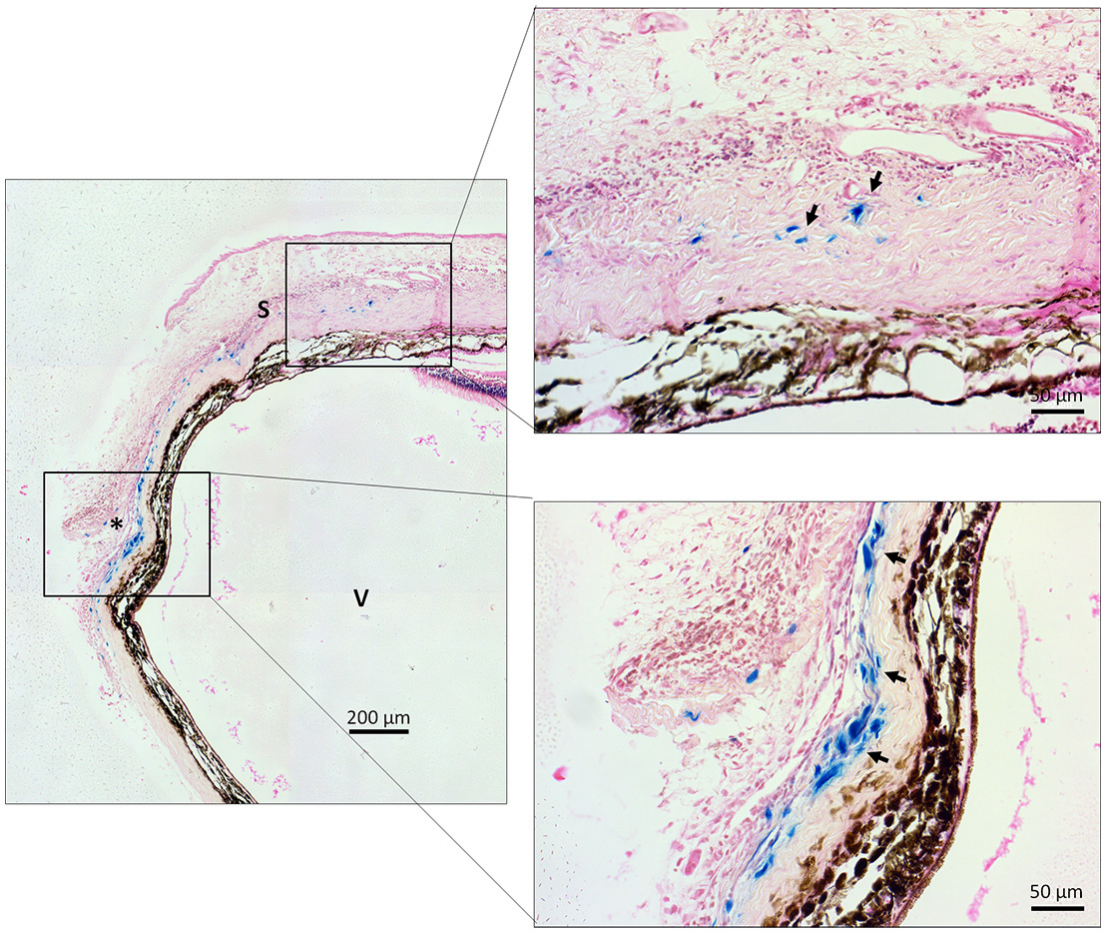
Local ocular distribution of adenovirus after a subconjunctival injection in the rats. Histological sections of the rat eyes injected with Ad-LacZ (1x10^10^ GC/eye) in the subconjunctiva. The blue staining (arrows) in the image indicate the expression of β-galactosidase. V: vitreous; S: sclera. Scale bar = 200 μm (left) and 50 μm (right).

Although bioluminescence imaging suggested that the adenovirus could spread out across the whole eye, it is of importance to localize the distribution of the adenovirus administered from conjunctiva. We therefore evaluated the local distribution of subconjunctival injection via histological analysis by using adenovirus encoding β-galactosidase (Ad-LacZ). As shown in Fig 3, 7 days after the Ad-LacZ injection (1x10^10^ GC/eye), the β-galactosidase expression extended along the adenovirus injection site toward anterior and posterior segments of the eye. The results indicated that adenoviral vector administered from conjunctiva could potentially spread from local injection site to other parts of the eye tissues.

### Quantitative assessment of local ocular distribution

Seven days after rats administered with Ad-hrGFP or PBS via subconjunctival injection in one eye, the eyes were enucleated and dissected into sclera, choroid, retina, and cornea immediately for evaluation of viral distribution of ocular tissues. The viral genomic DNA was detected in all ocular tissues (Table 1, n = 3) including the cornea from one rat (2578 GC/μg of total DNA), retina from one rat (3553 GC/μg of total DNA), choroid from two rats (3403 and 7359 GC/μg of total DNA), and sclera from three rats (108983, 49021 and 142618 GC/μg of total DNA).

**Table 1:** Evaluation of ocular distribution and local inflammatory response after a subconjunctival injection of adenoviral vector in the rats.

Ocular issues of rats injected with Ad-hrGFP	Ad-hrGFP DNA^a^	Ad-hrGFP mRNA	Increase of TNF-α expression^b^
**Sclera**	3/3 (108983, 49021 and 142618 gc)	3/3	0/3
**Choroid**	2/3 (3403 and 7359 gc)	2/3	2/3
**Retina**	1/3 (3553 gc)	1/3	1/3
**Cornea**	1/3 (2578 gc)	2/3	2/3

^a^Numbers in round bracket indicates viral genome copies per μg of total DNA of the tissue

^b^More than two fold of increase in comparison with PBS injected rats.

To further confirm the viral distribution and local immune response, the expression of transgene (hrGFP) from adenoviral vector and pro-inflammatory cytokine, TNFα were evaluated by quantitative PCR for detecting mRNA expression. As shows in Table 1, the cornea from two rats, retina from one rat, choroid from two rats, and sclera from three rats were found having expression of hrGFP mRNA. Moreover, the two fold expression of TNF-α mRNA was found in the cornea from two rats, retina from one rat, and choroid from two rats in compared to the PBS injection group.

### Assessment of systemic immunogenicity

**Fig 4:**
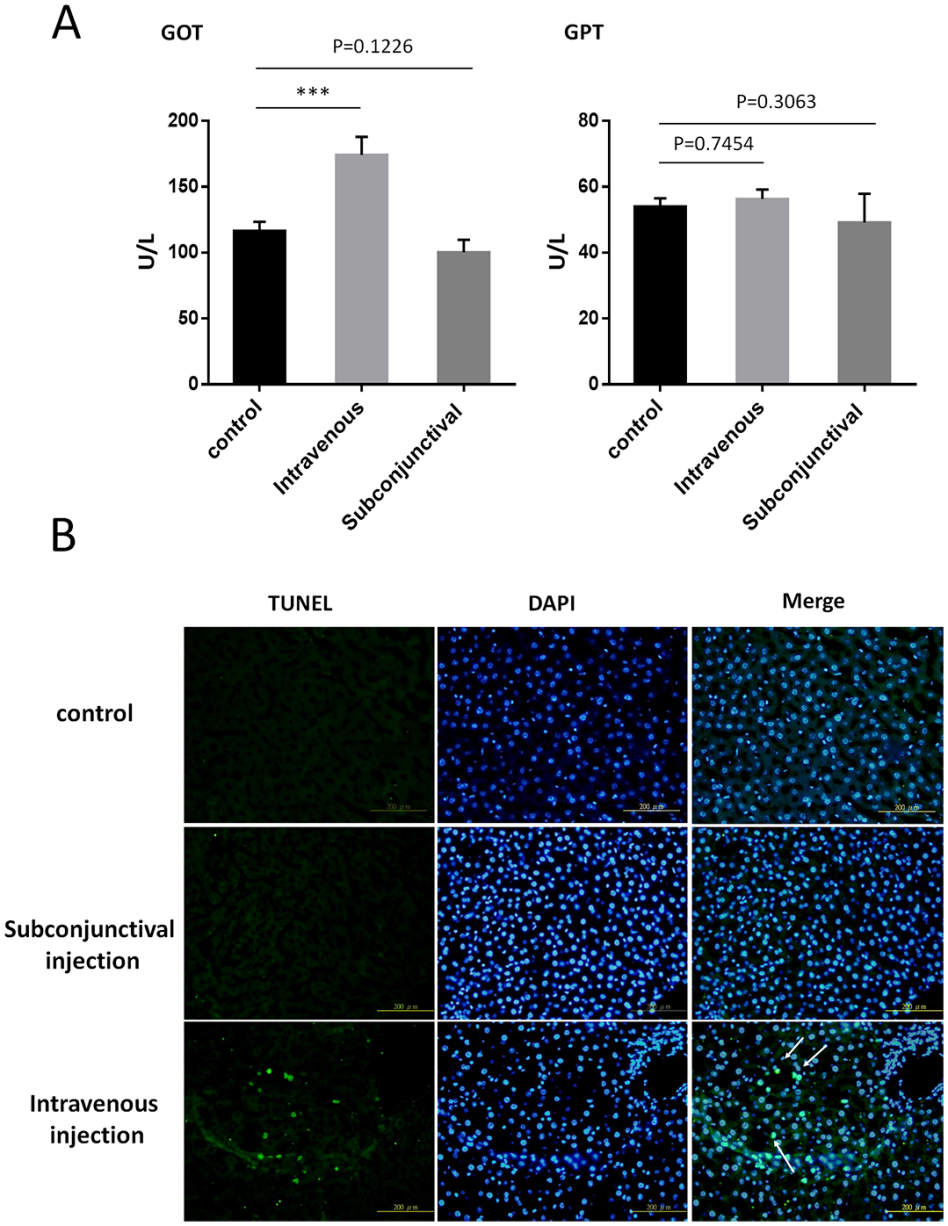
Effect of liver function in the rats received a single injection of adenoviral vector via different administration routes. (A) Serum level of glutamic oxaloacetic transaminase (GOT) and glutamic pyruvic transaminase (GPT) were measured at 14 days after an intravenous or subconjunctival injection of Ad-Luci (1x10^10^ GC/eye). Data are presented as the means ± SEM (***: p<0.0001, n = 6). One-way ANOVA followed by a Tukey’s test. (B) Sections of liver tissue were stained for TUNEL (green) and DAPI (Blue) 14 days after injection of adenovirus through different administration route. Scale bar = 200 μm.

Unlike the intravenous route, there is no significant change of GOT levels in the serum of the rats after administrating high dose of Ad-Luci (1x10^10^ GC/eye) via subconjunctiva injection (control: 116±6 U/L, n = 6; intravenous injection: 174±12 U/L, n = 6; subconjunctival injection: 100±8 U/L, n = 6; p<0.01; Fig 4A). The change in GPT level in the serum of three groups was also not significantly different (control: 54±2 U/L, n = 6; intravenous injection: 56±3 U/L, n = 6; subconjunctival injection: 49±7 U/L, n = 6; Fig 4A). Similarly, there was no significant increase of positively apoptotic cells in the liver of rats administrated with Ad-Luci (1x10^10^ GC/eye) via subconjunctival injection in TUNEL assay (Fig 4B).

**Fig 5:**
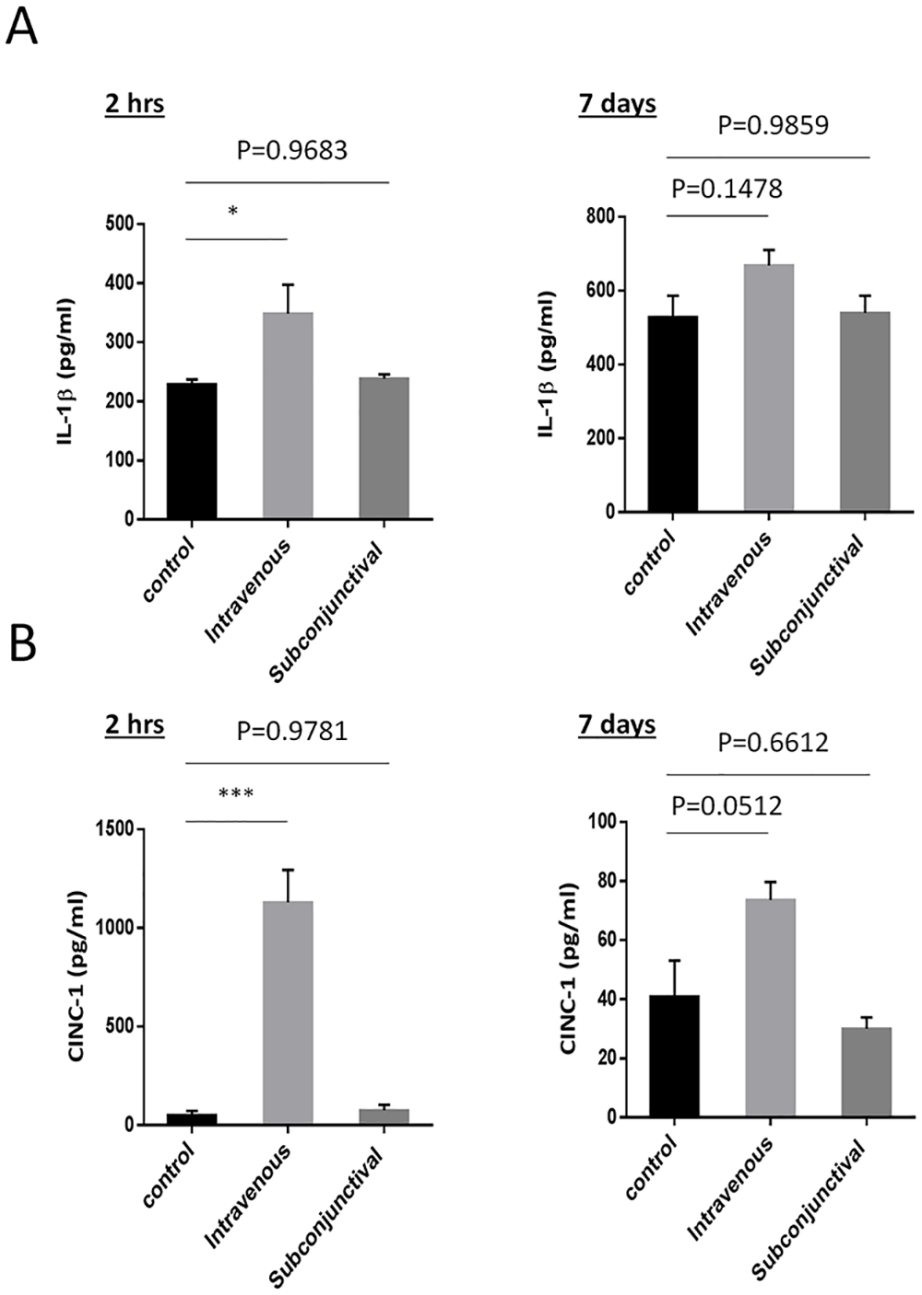
The serum level of IL-1β and CINC-1 in the rats received a single injection of adenoviral vector via different administration routes. Serum level of interleukin-1β (IL-1β; A) and cytokine-induced neutrophil chemoattractant factor-1 (CINC-1; B) were measured at 2 hours and 7 days after an intravenous or subconjunctival injection of Ad-Luci (1x10^10^ GC/eye). Data are presented as the means ± SEM (*: p<0.05, ***: p<0.0001, n = 6). One-way ANOVA followed by a Tukey’s test.

Adenoviral vector has been well known to elicit the activation of host immunity [18]. We therefore examined the pro-inflammatory cytokine at actuate phase of 2 hours and 7 days after viral injection. Serum level of IL-1β and CNIC-1 increased rapidly at 2 hours after intravenous injection of Ad-Luci (1x10^10^ GC/eye) and then settled after 7 days (Fig 5A and 5B). In contrast, there was no significant difference in terms of serum level of IL-1β and CNIC-1 between non-treated and adenovirus-treated rat via subconjunctival administration (Fig 5A and 5B). Therefore, unlike with intravenous administration, our data suggested that subconjunctival delivery of adenovirus is a safe delivery route that does not cause liver injury or elevate systemic immune responses.

### Retinal function analysis by ERG

To further explore the safety of adenoviral gene delivery via subconjunctival injection, the ERG was employed to evaluate the retinal function in the rat. There were no significant alterations found in ERG parameters (including a-wave amplitude, a-wave latency, b-wave amplitude and b-wave latency) between rats administered adenovirus via intravenous and subconjunctival injection 14 days later when compared to the baseline (Table 2). These results suggested that both subconjunctival and intravenous administration of adenoviral gene delivery do not lead to an adverse effect on retinal function.

**Table 2:** Analysis of retinal function by electroretinography (ERG).

Electroretinogram parameters	Control		p value	Intravenous		p value	Subconjunctival		p value
	Day0	Day14		Day0	Day14		Day0	Day14	
**a-wave amplitude, μV**	168.9±53.47	165.8±42.11	0.456	169.4±43.77	167.2±39.26	0.35	153.5±33.96	163.2±42.36	0.92
**a-wave latency, ms**	19.5±4.87	16.53±2.99	0.203	19.75±4.25	17.17±4.23	0.076	20.18±4.81	16.89±3.83	0.285
**b-wave amplitude, μV**	328.2±58.21	298.4±64.12	0.067	354.9±44.73	315.2±64.33	0.108	323.3±35.35	285.4±75.29	0.399
**b-wave latency, ms**	55.45±4.32	57.09±4.20	0.325	60.31±4.87	57.04±3.89	0.061	58.27±6.63	56.11±6.37	0.455

## Discussion

Adenovirus provides many advantages over the more commonly investigated gene therapy viral vector, AAV, especially in situations where large genes need to be delivered, or only transient gene expression or knockdown is required [9]. Adenovirus as a non-integrative vector, with 36-kilobase genome, grants the large space for therapeutic gene, which is particularly desirable for a rapid and robust activity of the transgene. Similarly, subconjunctival injection provides additional benefits over traditional intravitreal or intravenous gene delivery routes; it is much less invasive and has been shown in previous animal studies to be a safe and efficient mode of gene therapy for different ocular diseases [19–21]. Despite this, the use of subconjunctival injection has been thought to be largely restricted to treatment of anterior segment eye diseases [22–24]. Our study is, to our knowledge, the first that accurately characterizes the local distribution and safety profile of adenovirus administered via subconjunctival injection in rat eyes. The results of this study are highly significant clinically for they demonstrate that adenovirus via subconjunctival injection holds great potential for use in both anterior and posterior ocular disorders, especially those requiring short-term therapy, and that it is a safe form of gene therapy which does not cause liver injury or systemic immune activation.

Our study showed that a single subconjunctival injection of Ad-Luci leads to a peak expression of the reporter gene within 24 hours, but this expression gradually subsided in the following 64 days, and the low transgene expression could potentially sustain up to 16 weeks (Data not shown). Previous studies have shown that the duration of transgene expression mediated by adenovirus in animal eyes is only one month [25, 26] that due to the activation of the cytotoxic T-lymphocyte-mediated immunogenicity against the adenoviral-transduced cells, leading to the inflammation and short-term transgene expression [27, 28]. This brings up the concern that whether long-term expression of the transgene delivered in the eye could be obtained by an adenoviral vector. Repeated injection of adenoviral vector is assumed capable of sustaining the desired titers in the target cells. A study conducted by Hamilton *et al* suggests that repeated administration of adenoviral vector into the eye through the subconjunctiva injection is feasible and do not lead to an immediate immune response resulting in eliminated the transgene expression [29]. Moreover, a few other studies utilizing a helper-dependent adenovirus through modification of serotype also allow long-term expression of the transgene up to one year [25, 30]. These studies provide the possibility of long-term expression of the transgene delivered by an adenoviral vector in the eye either by repeated injection or using helper dependent adenovirus. In this regard, however, future studies maybe required to explore the assumption.

Our results were also consistent with previous studies that showed that the bio-distribution of adenovirus via subconjunctival injection is markedly limited to the eye [13]. Importantly, our study is the first to accurately characterize using qPCR which portions of the eye the adenovirus infiltrates when administered through subconjunctival injection, and the specific amount of genomic DNA from adenovirus disseminated into each of these different ocular tissues. In comparison with saline injected rats, all four portions of ocular tissue from the rats that received Ad-hrGFP subconjunctivally contained adenoviral genome copies of hrGFP at dramatically different levels. The sclera contained the highest level of adenoviral genome copies in all three rats that received subconjunctival Ad-GFP (108983, 49021, and 142618 GC/μg of total DNA of sclera). In two rats, adenoviral genome copies were also found in the choroid, which contained the second highest level of genome copies. However this level was far less than in the sclera (3403 and 7359 GC/μg of total DNA). Only one rat had adenoviral genome copies in the retina and cornea, where the levels of genome copies were the least (3553 and 2578 GC/μg of total DNA respectively). Our results suggest that adenovirus is able to infiltrate into the different tissues across the eyeball over time, including the posterior ocular segment, and therefore genes may potentially be distributed by this route for disorders of the back of the eye.

Our study also showed that the amount of adenovirus infiltrating into the ocular tissues varies across the different portions of the eye. Subconjunctival injection is believed to be an administration route that avoids the conjunctival barrier, allowing more access for drugs to diffuse into sclera [31]. Additionally, choroid plexuses and the blood-retinal barrier that prevents drug compounds entering into the retina and cornea are much harder to penetrate than sclera [32]. These properties likely explain why the largest amount of adenovirus was found in sclera, followed by choroid, retina and cornea.

One of the reasons for the limited use of adenovirus as a vector in gene therapy so far is that it has been previously shown that adenovirus elicits local or systemic immunogenicity [33, 34]. Previous studies have demonstrated that adenovirus can induce TNF-α, a critical inflammatory factor taking part in immune response [35, 36]. In this study, appears to be local immunogenicity induced by subconjunctival administered adenovirus mild, when compared to the intravenous route, and short-lived. The local inflammation disappeared from the sclera where the injection was given and shifted to other parts of the eye 7 days after a single injection. Additionally, the ELISA results suggested that the serum level of IL-1β and CNIC of rats administered with adenovirus via subconjunctival injection were the same as those in non-treated rats both 2 hours and 7 days after injection, while a significantly higher level of these two inflammatory factors was observed in the rats receiving adenovirus intravenously. Similar findings were discovered by Wen and colleagues who reported that the acute inflammatory response induced by adenovirus gradually decreased within two weeks on the basis of histological analysis, and that the inflammatory response was confined to the conjunctival injection site [13]. Another clinical study found that only a mild local immune response without evident systemic immunogenicity resulted from adenovirus after intraocular injection [37]. Therefore, with this evidence and our findings, we believe the administration of adenovirus in the eye would only elicit a mild local immune response that is temporary, and would not result in systemic immunogenicity.

Retinal function analysis by the ERG suggested that subconjunctival injection of adenovirus did not cause any adverse effects. Other studies have also demonstrated that photoreceptor function has not changed when inflammatory cells infiltrated in the choroid after subconjunctival injection of adenovirus [38]. Thus, there is the potential for adenovirus delivered by subconjunctiva to be used safely and effectively to treat posterior segment ocular diseases.

The ideal route of administration for gene therapy is one that achieves good local effect in the target tissues with minimal systemic toxicity. Previous studies have demonstrated that adenovirus can circulate to the liver and spleen after subconjunctival injection in different animals, but at a very low level in terms of adenoviral genome copies [13, 21]. It is not clear to date whether such low level of adenovirus could have an unfavorable effect on the liver function after subconjunctival injection. Our data from the liver function analysis, including measurement of GOT and GPT level in the serum and TUNEL staining, implicated no hepatic toxicity in the rats after Ad-Luci administration through conjunctiva. Therefore, subconjunctival administration of adenovirus appears to be a useful mode of gene delivery as it is expressed locally in the eye, with minimal systemic effects.

In conclusion, this study has characterized the local distribution of the adenovirus in various ocular tissues across the eyeball after subconjunctival administration. This opens the door to the use of adenovirus as a gene therapy vector in disorders of both anterior and posterior eye segments eye diseases. This study also demonstrated that adenovirus injection via subconjunctiva, when compared to intravenous administration, is an efficient and safe route for gene delivery in the short term, with no effect on retinal function, no systemic toxicity, and only minimal local and systemic immunogenicity. Ultimately, our study exemplifies that adenovirus delivered via the subconjunctival route is a promising safe and effective mode of gene delivery for many ocular diseases requiring short-term gene therapy.

## Supporting Information

S1 Fig Timeline of the study to characterize the adenovirus-mediated gene delivery via subconjunctival administration. (TIF)

S2 Fig Standard curves of adenoviral genome copies for quantitative PCR standards. (TIF)
