# Supplementary figures and images for: Gene Delivery by Subconjunctival Injection of Adenovirus in Rats: A Study of Local Distribution, Transgene Duration and Safety
Source: PLoS One. 2015 Dec 7;10(12):e0143956. doi: 10.1371/journal.pone.0143956 (PMC4671571; doi:10.1371/journal.pone.0143956)

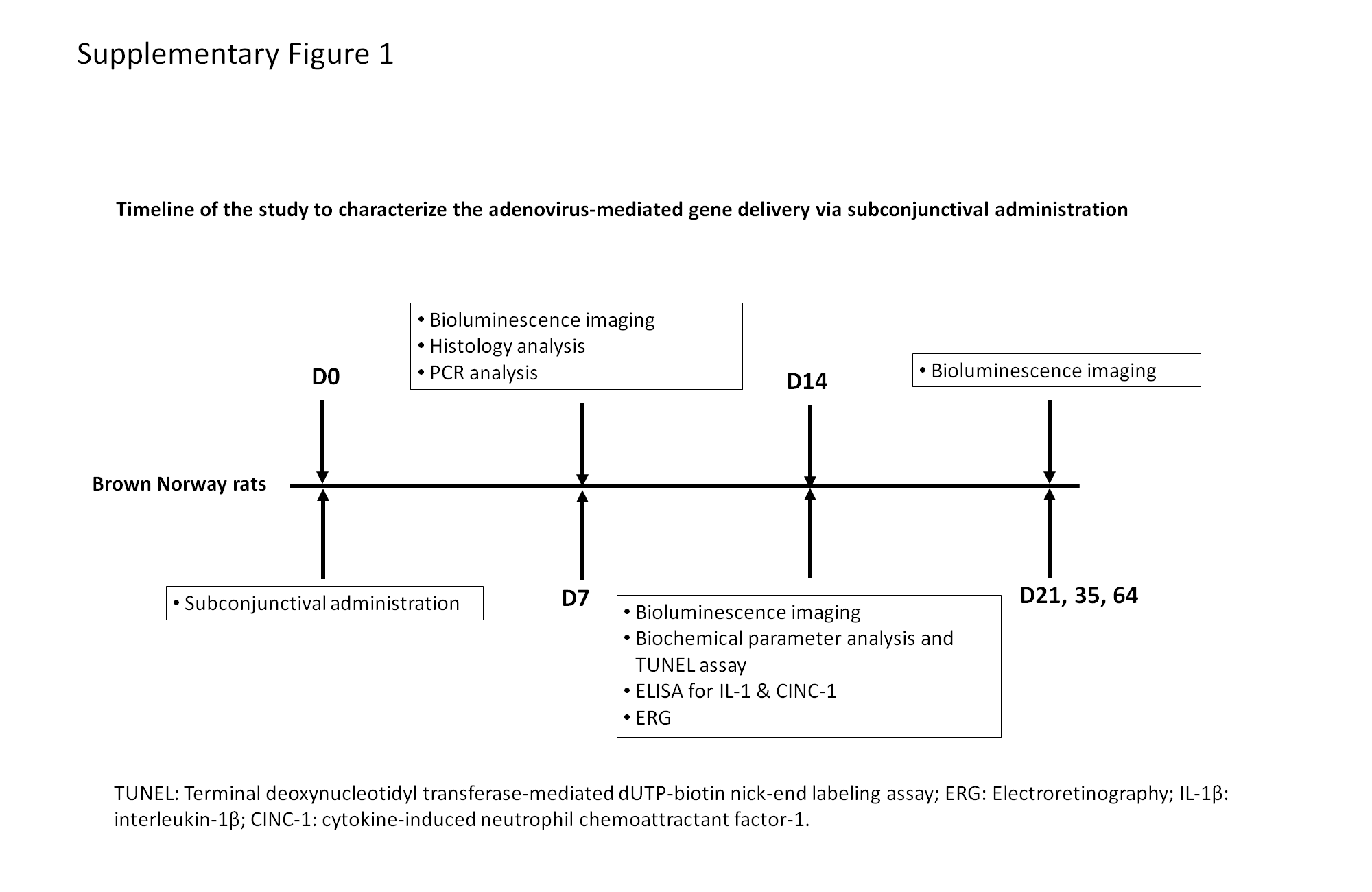

Supplement: S1 Fig — (TIF) [file pone.0143956.s001.tif]

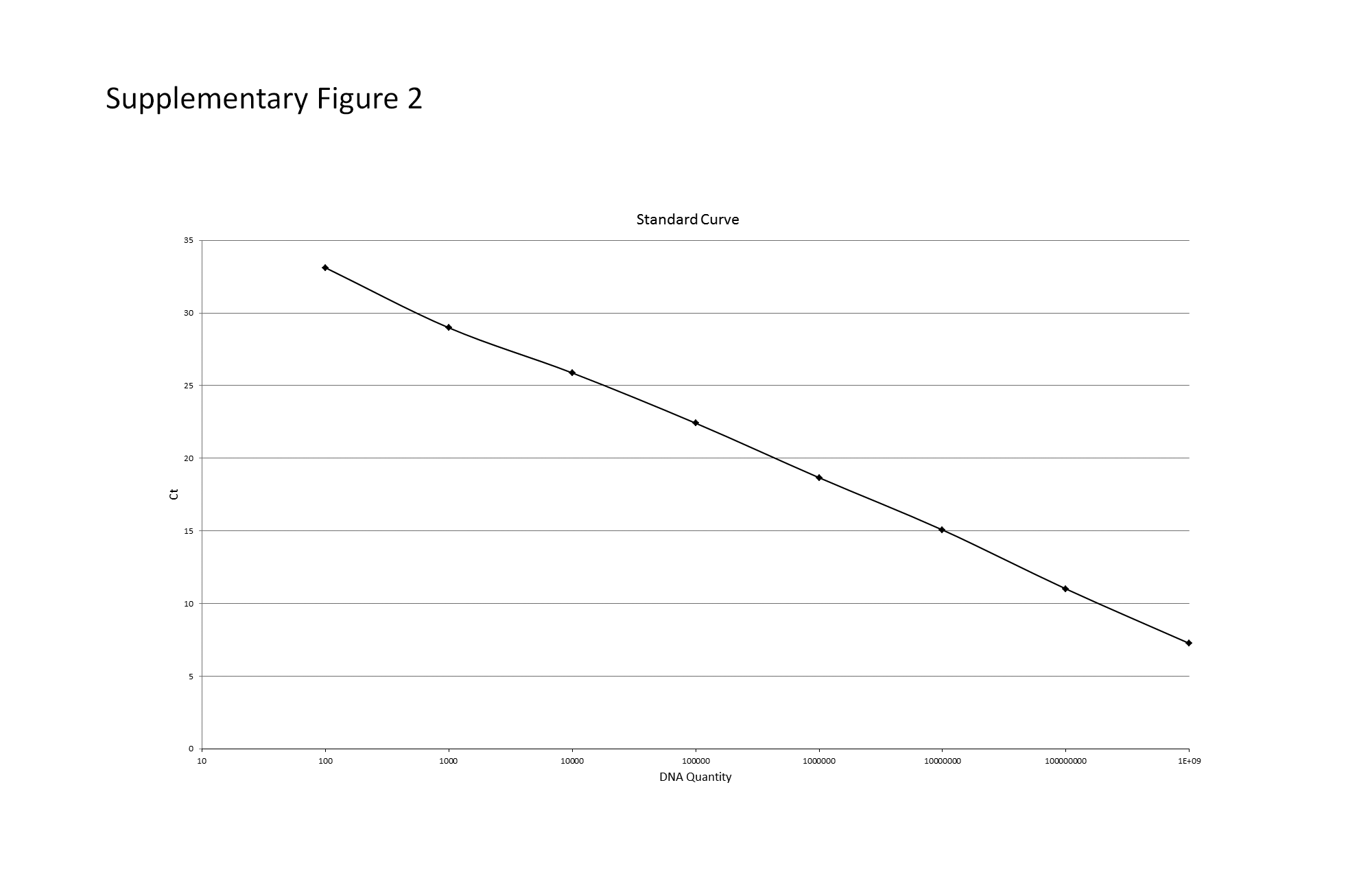

Supplement: S2 Fig — (TIF) [file pone.0143956.s002.tif]
